# Ethics in Community-University-Artist Partnered Research: Tensions, Contradictions and Gaps Identified in an ‘Arts for Social Change’ Project

**DOI:** 10.1007/s10805-016-9257-7

**Published:** 2016-04-07

**Authors:** Annalee Yassi, Jennifer Beth Spiegel, Karen Lockhart, Lynn Fels, Katherine Boydell, Judith Marcuse

**Affiliations:** 1 School of Population and Public Health, University of British Columbia, 430-2206 East Mall, UBC, Vancouver, BC V6T 1Z3 Canada; 2 International Centre for Art for Social Change and Faculty of Education, Simon Fraser University, Burnaby, BC Canada; 3 English Department, Concordia University, Montreal, QC Canada; 4 Black Dog Institute, University of New South Wales, Sydney, Australia; 5 Departments of Psychiatry and Dalla Lana School of Public Health, University of Toronto, Toronto, ON Canada

**Keywords:** Ethics in teams, Collaboration, Community-university-artist partnered research, Interdisciplinarity, Team dynamics

## Abstract

Academics from diverse disciplines are recognizing not only the procedural ethical issues involved in research, but also the complexity of everyday “micro” ethical issues that arise. While ethical guidelines are being developed for research in aboriginal populations and low-and-middle-income countries, multi-partnered research initiatives examining arts-based interventions to promote social change pose a unique set of ethical dilemmas not yet fully explored. Our research team, comprising health, education, and social scientists, critical theorists, artists and community-activists launched a five-year research partnership on arts-for-social change. Funded by the Social Science and Humanities Research Council in Canada and based in six universities, including over 40 community-based collaborators, and informed by five main field projects (circus with street youth, theatre by people with disabilities, dance for people with Parkinson’s disease, participatory theatre with refugees and artsinfused dialogue), we set out to synthesize existing knowledge and lessons we learned. We summarized these learnings into 12 key points for reflection, grouped into three categories: community-university partnership concerns (*n* = 3), dilemmas related to the arts (*n* = 5), and team issues (*n* = 4). In addition to addressing previous concerns outlined in the literature (e.g., related to consent, anonymity, dangerous emotional terrain, etc.), we identified power dynamics (visible and hidden) hindering meaningful participation of community partners and university-based teams that need to be addressed within a reflective critical framework of ethical practice. We present how our team has been addressing these issues, as examples of how such concerns could be approached in community-university partnerships in arts for social change.

I’ve rarely heard of a collaboration involving teams made up of artists, researchers, community participants and partners that has gone smoothly…

– artist-activist in our collaborative project

What ethical concerns, challenges, and tensions emerge when a multi-partnered interdisciplinary research team embarks on a five-year initiative? How might team members and partners learn to ethically navigate these? What key ethical considerations and actions need to be highlighted to inform and assist those engaged in post-secondary teaching and/or research? Historically, attention to ethics in research stemmed from the biomedical field, where, for instance, medical experiments were conducted on prisoners without their consent (Becker 2005). However, ethical issues in diverse research areas have received increasing focus in recent years (Norris et al. 2007; A. Yassi et al. 2013; Stenmark et al. 2010; Reid and Brief 2009). For example, in aboriginal health research, attention has been drawn to adhering to the 4 R’s: respect, reciprocity, relevance and responsibility (Canadian Institutes of Health Research 2007; Glass and Kaufert 2007; Kirkness and Barnhardt 2001), with funding bodies introducing guidelines to prompt researchers to carefully attend to ethical concerns (Canadian Institutes of Health Research et al. 2010). Discussions of ethical issues in global health research have also recently emphasized the responsibility for social justice and solidarity, including the ethical imperative to build local capacity (A. Yassi et al. 2013; Benatar and Singer 2010). Similarly, ethical issues in community-engaged research have attracted attention (Khanlou and Petera 2005; Reid and Brief 2009; Tuck 2009; Tuck and Yang 2014) and guideline development.

Meanwhile, there has also been growing interest in arts-based research (SM. Cox et al. 2010; Boydell et al. 2012), and a committee of research funding bodies in Canada have called for guidance in this area (Blackstone et al. 2008). There have been workshops on ethics in use of visual art resulting in recommendations (S. Cox et al. 2014) calling for addressing concerns about authorship and ownership, minimizing harm, consent, confidentiality, representation and audience, as well as what has been called “fuzzy boundaries”(Gubrium et al. 2014) or the blurring of roles and purpose. Notwithstanding this attention to ethical issues associated with *arts*-*based research methods* for data gathering or for disseminating messages, such as photovoice, murals, poetry, dance and theatre (SM. Cox et al. 2010; Fraser and al Sayah 2011; Boydell et al. 2012; S. M. Cox and Boydell 2015), there has been no substantial discussion of ethical issues in quantitative or mixed-method studies to “evaluate” arts-for-social change (ASC) programs in multi-institutional community-university-artist collaborations. Researchers have helped focus the debate by recognizing that there are what some researchers call “big E” or procedural ethics, as opposed to the everyday “small e” or the everyday micro-ethical issues that inevitably arise (Komesaroff 1995; Guillemin and Gillam 2004). Whilst considerable progress has been made in recent years to help guide some of the thinking, many pertinent issues flagged to date have not yet been fully unpacked let alone critically theorized. Moreover, no synthesis exists of how the various ethical issues interrelate.

Our research team, comprising critical theorists, artists, health researchers, education specialists and social scientists, as well as community-activists working in non-governmental organizations (NGOs), government and the private sector, were co-applicants or collaborators on a grant from the Canadian Social Sciences and Humanities Research Council (SSHRC) to launch a research partnership on arts-for-social change. Based in six universities and involving over 40 community-based individuals and organizations, our team launched this five-year research program to: 1) study the teaching and learning about ASC; 2) investigate current practices of evaluation of ASC projects and explore ways to improve these; and 3) develop tools and share effective collaborative practices for partnership capacity building. In the context of our project, which we abbreviate as “ASC!” we are particularly focused on the area of ASC very broadly defined as art that is created collectively by groups of people (who may not self-identify as artists) about what matters to them, through arts or dialogic processes that are facilitated by an artist or group of artists (Art-for-social change research team 2016). All team members brought their own ASC experiences and/or pedagogical and research practices to the project. Additionally five main field studies (social circus with urban youth; dance with people with Parkinson’s disease; theatre with people with disabilities; participatory theatre with refugees; and arts-infused dialogue) were funded as part of ASC! to fuel our exploration. In embarking upon our research, we encountered many new ethical dilemmas for which we were unable to find resources on best practices. We therefore set out to critically synthesize existing knowledge and combine it with reflections on issues that arose during the first half of the project, so as to inform others who launch such research partnerships. Our hope is that those engaged in post-secondary research and education might share our learning so that the complexities and difficulties that we experienced in our multi-partnered, interdisciplinary project might be anticipated and addressed in a proactive rather than reactive fashion. Discussing these topics as matters to be addressed within a framework of considering ethical issues may avoid needless tensions that could undermine the research.

## Methods

We undertook a reflexive process to analyze the issues identified from our own experience. Reflexivity in research involves “reflection on self, process, and representation, and critically examining power relations and politics in the research process, and researcher accountability in data collection and interpretation” (Sultana 2007). Our reflections and musings built on the work of others, for example Guillemin and Gillam (2004), who proposed that reflexivity is a helpful conceptual tool for understanding both the nature of ethics in qualitative research and how ethical practice in research can be achieved. Our team also drew upon the work of Barry and her colleagues (Barry et al. 1999) who position reflexivity as a team activity rather than an individual one. Our identification of the unique ethical issues encountered in the ASC! projects were enhanced by attending to ‘what is going on while researching’ (Koch and Harrington 1998) and this, in turn, enriched our analysis and theoretical thinking. Similar to Michalos (2001), we acknowledge that there are a wide variety of perspectives about ethics and morality, with no one best structure to guide moral decision-making. As such, being a diverse interdisciplinary group, we made no attempt to choose one moral code to guide our selection of what constituted an ethical issue but rather tacitly embraced what Michalos characterized as two basic normative considerations involved in most moral theories, namely, imperatives “which, if followed, would bring about general human well-being”, and “do not permit the agent to make an exception in his own favour”. Any issue the team agreed met these two criteria was included within this study.

Once we identified the matters of concern that we had experienced and considered to be “ethical issues”, we formulated key themes then grouped these into categories; the three broad groupings of ethical issues that emerged were community-based concerns, arts-specific dilemmas, and team-related issues. Next, to gather additional input from our large team of nine co-investigators and 44 collaborators, we created a questionnaire for anonymous completion online or in paper-based form; this ethics questionnaire helped further our understanding of those issues that we as authors had identified as matters of concern. We obtained ethics approval for this study from the University of British Columbia Behavioural Ethics Review Board under certificate number H14-01041.

## Results

Table 1 summarizes the ethical issue identified in the survey combined with reflections from our own experience as researchers, and the approach we are taking for addressing these. Each of these is elaborated upon below.

**Table 1:** Summary of ethical dilemmas and possible reflective approaches to resolutions we identified

Ethical dilemma	Description	Possible approach
1. Ethical issues related to community-university partnering		
1.1 Ethics of meaningful participation	Community partners in organizations with limited funding, as well as independent artists engaged with academics in collaborative research, have to volunteer their time to participate in the research, while those hired for the research, or leading the research based at universities are funded for their participation; this situation creates a power imbalance in ability to participate in a research partnership.	Research teams need to be sensitive to this economic imbalance and seek creative solutions that do not violate funding rules; our experience suggests that means can be found to provide at least partial remuneration for research contributions while keeping within guidelines. Simultaneously, funding rules need to be challenged so as to address current funding inequities.
1.2 Ethics of consent	There are constraints as to who can easily provide consent (e.g., only those over 18; people with enough literacy and time to read detailed text-heavy materials imposed by Research Ethics Boards (REBs) and who are not considered about signing documents for other reasons such as illegal presence in the country; these constraints can result in excluding important components of the populations, if taken too literally. Conversely individuals declining consent is important to document as well, analyzing why this refusal is occurring.	Consent needs to be made context-sensitive and flexible to avoid excluding the voices of marginalized populations while at the same time carefully respecting the rights of individuals and communities to refrain from engaging in the research process; reflecting on underlying socioeconomic and political power dynamics may help explain reluctance and indeed refusals of individuals to participate.
1.3 Ethics of raising false expectations	Community partners often expect faster turn-around times in output production than research allows; it Is essential that the community partners understand the time-frame to output production as well as limits of the research to avoid false expectations.	Good communication is essential at the outset to ensure clear understanding of priorities/needs of all involved in terms of time-frame and impact; researchers must responsibly ensure that the community partner is clearly aware of the limitations of the study with respect to the speed at which research outputs would be produced, what questions would be answered, and what, if any, the likely impact of the research will be with respect to social change, as participants define it.
2. Issues related to the inclusion of Arts-Based Research and Research in the Humanities		
2.1 Ethics of stifling creativity in participatory action research: protocol rigidly hampering artistic process	Sometimes unanticipated opportunities arise that are desirable to pursue, e.g., opportunity to film an artistic process (with consent of participants), or conduct informal interviews at an arts show, training session or other community event; strictly speaking, if ethics approval has not been obtained in advance, this is not supposed to be done – but would constitute an unfortunate lost opportunity to improve the quality of the research.	Guidelines for ethical practices are essential but these should leave room for flexibility to accommodate the unpredictable nature of community-engaged arts-based research that may present important research opportunities. Here, attention to procedural ethics could be framed as in a dialectical relationship with microethics, so that ethical principles guide reflective practice.
2.2 Ethics of authorship and ownership of arts-based intervention products	Creative collaboration leading to the generation of art raises questions relating to authorship and ownership of the work; consent to use the work; and the truthfulness or adequacy of the work as a representation of participants’ experiences.	Researchers should be encouraged to create written agreements about ownership within the research team and with participants that reflect the needs and sensibilities of all. This could be approached as a procedural ethical issue, with flexibility to reflect and adapt as conditions require.
2.3 Right of acknowledgement versus protection of anonymity	There is a tension between the goal of anonymity and protecting vulnerable participants on one side, and the desired goal of stigma reduction strategies that promote speaking out. Further, participants sometimes want their identity to be known, as they are proud of their contribution and want their insights offered to the researchers to be openly attributable to them.	If future consequences are properly explained in a manner that is understood by participants, they should be allowed to determine whether or not they want to be identified and should have the right to change their position during the research period.
2.4 Ethics of dangerous emotional terrain	In ASC projects, the goal is often to push participants beyond their typical comfort zones and expose them to varied perspectives and experiences in a meaningful manner; there arise ethical challenges associated with the danger of encountering difficult, emotionally charged, risky and traumatic issues, as well as “undoing” participants’ previous conceptions.	Artist/researchers should have permission to explore emotionally-charged topics as long as they are trained to deal with potential problems and have informed support available for everyone involved including the artists/ researchers, themselves; training is key. Moreover, researchers should be cognizant that their own values will inevitably influence the process and care must be taken to avoid imposing dominant values.
2.5 Ethics of representation	Misrepresentation of art can easily happen, and there are ethical implications of divergent interpretations.	Taking preliminary “results” back to artist participants for feedback would be a good practice. Another is to establish a monitor for the group during the interpretation process.
3. Team issues		
3.1 Ethics of caring for team members, students and staff	Ethics of team relations – and valuing all team members through taking care of their material and emotional needs - is part of the imperative of the research.	Always keep the wellbeing of all team members, and team dynamics in the forefront, being willing to challenge institutional norms if necessary.
3.2 Ethics of researcher engagement and commitment	Different researchers shoulder different responsibilities and contribute different levels of commitment, and time. Sometimes these are known from the start; sometimes work or family commitments outside the project impact on originally envisioned commitments; this creates dilemmas for other team members and partners.	Time commitments should be made transparent to all at the beginning and reviewed periodically; regular “check-ins” need to be planned to take unforeseen circumstances into account.
3.3 Ethics of expanding the team after the project is in process	Research is increasingly becoming a collective endeavor, and often researchers and artists seek to co-research and co-create with colleagues, friends, and those with whom there is a shared recognition of theoretical resonance, expertise, perspective and previous lived experience, sometimes only discovering this resonance after the project has started.	Clearly stated criteria for inviting colleagues and partners should be articulated at the beginning of the process; bringing in new expertise must be undertaken with dialogue with original team members to ensure that the new recruit would not undermine the complex research dynamics in any way.
3.4 Ethics of interdisciplinarity – different cultures of publication, collaboration, and notions of ethical research	There are very different cultures in different disciplines, such that what in unethical in one discipline might be the norm in another – with respect to norms in publishing, extending invitation to co-author, how collaboration is conducted and indeed what constitutes ethical research practice.	Teams of artists, community activists and scholars from different disciplines, must openly discuss the varied needs and expectations with respect to authorship, collaboration and the implications of one approach to research on the integrity for another. Critical theorists, arts-based researchers, qualitative and quantitative research do not easily mesh – therefore mixed methods studies attempting to embody both approaches need to engage in considerable dialogue and reflection at all stages of the research.

### Community-University Partnership Issues

#### Ethics of Meaningful Participation

There is considerable scholarship on the desirability of fully involving research participants in processes of knowledge creation (Kitson et al. 2013; Lencucha et al. 2010). Indeed to help secure research funding, university-based researchers often solicit letters from community-based partners attesting to their willingness to play a meaningful role; this was indeed required in the case of our project, funded specifically from SSHRC’s “partnership” research program. Many participatory and feminist methodologies emphasize non-hierarchical interactions and bi-directional learning, carefully considering how research questions are framed and how data collection methods may be embedded in unequal power relations between researchers and participants (Sultana 2007; Bondi 2003). We found that a barrier to equity arose when an overly rigid interpretation of SSHRC’s rules was originally embraced that discouraged payment to community-based team members for their involvement in the research. In many settings within which we work, community members cannot participate in research activities with scholars unless they are financially compensated (for example, youth are often asked to take uncompensated time away from after-school employment such as shift work to participate in research activities related to ASC projects without receiving compensatory payment). Our research team was thus faced with an ethical dilemma of how to ensure that artists and community-based personnel were remunerated for their time in light of rules that suggested that partners in the research should be contributing to the research from their own resources, not being paid from the grant.

In one of our field studies ethical tensions arose when it became apparent that one of the NGOs we had invited to collaborate on the design and implementation of surveys and interviews with participants in their program had limited funding for staff time to do this work. The university researcher on our team who was working most closely with this NGO was uncomfortable asking the NGO to subsidize the substantial time necessary to provide meaningful assistance with the conceptual development and logistical support that the methodology required. Indeed, despite their keen interest in participating in the research, the board of directors of the organization would not permit the staff member to contribute this time without receiving funds from the grant to hire an assistant for that staff member, as without such assistance core basic service tasks of the organization could suffer. As some ASC! team members were concerned about establishing a precedent of funding community organizations – given the large number of community organizations involved in ASC! - one of the senior ASC! researchers, who had access to funds from a different source, provided remuneration to the NGO in question as an honorarium for this important research work.

The majority of respondents to our ethics questionnaire indeed echoed these tensions around financial access and equity necessary for meaningful participation. As such, the university-based researchers throughout the ASC! team felt an ethical responsibility to find ways to address the wide discrepancy in remuneration for time spent on research by academic versus community partners, so as to avoid, on one hand, the exploitation of partners who are asked to “volunteer” their limited time, or, on the other hand, disenfranchising those with least financial ability to participate. We were successful to varying degrees, given the context of individual field studies, the access individual researchers had to other funding, and our ability to allocate funds within the guidelines of the grant we had received. In reflecting on this dilemma, one of the respondents to our questionnaire referred to the unequal ability to participate as: “… *reflective of the way non*-*academic knowledge is devalued*.” Another stated: “*Financial reimbursement makes it possible for community partners to participate and feel like they are joining collaborations at an equal level*.”

Research funding agencies recognize the need to cover community-based research expenses, such as providing honoraria for those who are interviewed or participate in focus groups, as well as transportation costs, food and incidental costs, but often do not adequately recognize the financial costs associated with participating in the research incurred by community partners (especially individuals within these organizations who do not receive full-time salaries from their NGOs, or are independent artists). In our case, all our field studies sought to incorporate creative ways to remunerate artist and community team members, either as community-based research assistants, consultants, or through larger honoraria, and/or via other funding sources to complement the SSHRC funds. In an era of austerity in which NGOs have difficulty maintaining adequate funding for their activities, our experience in ASC! partnered projects has led us to conclude that it behooves academic researchers (and funding bodies) to find ways to ensure that there are no financial barriers to meaningful participation. We stress that while this dilemma may be resolved in different ways, and we are in no position to advise what process to employ in specific contexts, our work to-date strongly suggests that treating this issue as an ethical priority is indeed warranted.

#### Ethics of Consent

Procedural ethical considerations as assessed in REBs have to ensure that consent to participate is informed and voluntarily provided. As we found in our ASC! studies, critical researchers also need to ensure that *the process* of documenting informed consent does not inadvertently disenfranchise people whose voices need to be heard. Not everyone can easily provide informed written consent. For example, some REBs suggest that those under 18 should not be asked to sign consent without parental or guardian approval. Another example is obtaining consent from people do not have enough literacy in English to read detailed text-heavy materials; and yet another relates to the reluctance of some individuals to have a written record of their names on official documents (for instance, if they lack legal status in the country). In using digital storytelling with aboriginal youth in Northern Canada, Flicker observed that written consent forms were seen as tools of colonialism, and recommended a respectful culturally-appropriate process of verbal consent instead (Gubrium et al. 2014). A recent systematic review (Tamariz et al. 2012) found having a research team member speak one-on-one with study participants was the most effective strategy for obtaining informed consent, however how to document for REBs that this consent has been given still requires thought.

As our project included programs targeting youth with marginalized lifestyles—often living or working in the streets, and some under 18 but living on their own, as well as people with mental health issues, and those who may not yet be considered legally in the country —the need to obtain written evidence of informed consent to satisfy REBs was a concern. In our case, with respect to age, for example, in some settings we decided to allow youth to decide for themselves if they were eligible to participate as adults, and did not require disclosure of legal age. We recognize that this approach may be contentious and could lead to a slippery slope; we make no attempt to advocate for this approach for all contexts. Our point, as with the previous issue, is that critical thought is warranted on the issue of obtaining evidence of informed consent without threatening or disenfranchising oft-times marginalized voices. Importantly, Guillemin and Gillam remind us that it is in the researchers’ personal interactions with research participants “that the process of informed consent really occurs—not on the pieces of paper that an ethics committee peruses” (Guillemin and Gillam 2004).

From another perspective, Tuck and Yang discuss “inquiry as invasion”, arguing: “this invasion imperative is often disguised in universalist terms of producing ‘objective knowledge’ for “the public” (Tuck and Yang 2014). These theorists go on to highlight the critical nature of attention to refusal when it comes to projects involving the arts: “In teaching and learning refusal, we often turn toward art to give language to the intuitive. Using art to think/feel through theory - to decode power and uncode communities - trains our intuition. Refusal is not just a no, it is a performance of that no, and thus an artistic form.” Refusal is also an act of resistance and thus an act of agency. As such, research teams involving the arts would be well advised to appreciate the complexities of enabling those who want to speak to be heard, yet also documenting refusal to consent as itself an important statement. In the words of Tuck and Yang, we need to “stop touching the objects, and to observe instead the objectifying space and its sexual, racial, and biopolitical architecture” (Tuck and Yang 2014).

#### Ethics of Raising False Expectations

Through our review of the literature (Dyck and Allen 2013), as well as consultation with our partners, we identified that raising false expectations among participants and/or partners needs to be regarded as an ethical concern. Taking the time to conduct thoughtful research, with the reflection and careful logistic planning needed, is an ethical responsibility, one recognized by REBs. Nonetheless, once the research is underway, partners often are keen to receive the results of the research as quickly as possible, so that they might benefit from the results in their operational planning. At the same time, researchers may feel pressed to promise and/or agree to unrealistic timelines, under pressure from funding bodies and community partners alike. As such, while serious efforts need to be made to adhere to timelines, it is incumbent on researchers to ensure that all partners understand that the research process often takes longer than expected.

This ethical concern applies not only to timeframe issues but also to limitations of study design. Community partners in collaboration with researchers may have expectations of research outcomes beyond the parameters of what the research design could fulfil. For example, some designs may address the question of how a certain arts-based program functions, but not whether it results in improvements in certain specified outcome better than do non-arts programs. Our experience highlights the importance of ensuring that partners understand what questions will be able to be addressed by the research and which will not; identifying the limitations of the study as early as possible in the collaborations as well as frequently along the way; and providing realistic timelines as part of the comprehensive dialogue and consultation that is integral to any participatory project.

In addition, an ethical concern we have experienced arises from institutional and funder imposed time-consuming administrative and evaluative procedures that potentially interfere with ongoing research and program operations. Procedures that require researchers to submit onerous reports with constraining timelines could hinder rather than support ongoing research. If we are to consider the ethics of engagement between funding bodies and researchers as one of mutual respect, dialogue not only with community partners but also with funders could result in greater appreciation for the organic unfolding of research as experienced in the field. We have had several examples in our partnership in which our researchers inadvertently created unrealistic expectations; our experience suggests that treating the issue of mitigating false expectations as an ethical imperative indeed has merit. At the same time, some of our ASC! researchers have felt that their energies were sapped by requirements to attend to a myriad of bureaucratic requirements and obligations from home institutions and funding bodies.

### Issues Related More Specifically to Research About Arts Interventions

#### Ethics of Stifling Creativity in Arts-Infused Participatory Action Research

An issue identified in our reflexive discussions perhaps more specific to ASC intervention studies than to other types of community interventions, was related to REB requests for detailed descriptions of how research creations will evolve, a request, if too rigidly imposed, that could stifle creativity and undermine emergent learning. Interestingly, while some ASC! questionnaire respondents felt that it was not possible to outline how a research creation would develop or even articulate its precise aims, most ASC! respondents felt that creating a detailed description of potential outcomes and means of knowledge mobilization was not disruptive but rather “incredibly helpful”. One questionnaire respondent noted that REBs are evolving to recognize emergent research designs, as they do for research questionnaires and semi-structured interview guides, whereby what is sought in the research activities depends on what emerges. Some respondents also noted that REBs are indeed processing amendments to ethics approvals very quickly: “*Our University is very supportive of emergent research*, *and accepts questions as guidelines*, *not the actual ones. Amendments are easily followed through*, *in my experience*.”

Sometimes, however, as discussed by Cox and Boydell (2015) and as we experienced during our research, unanticipated opportunities arise during the study that are desirable to pursue (for example, an opportunity to film a performance or to conduct informal interviews at an arts event). Strictly, if a REB did not pre-approve research activities they should not be completed (regardless of the process used to obtain consent at, during or after the event). Clearly, this problem arises in any community and/or arts intervention that is emergent and subject to who is participating in the room. Flexibility and nimbleness are especially needed in studying ASC projects; therefore teams should indicate within their REB approval protocols that retroactive approval may be sought from partner organizations to permit researchers to pursue research opportunities presented by community partners and participants as they arise. Some REBs are reluctant to pre-approve activities, and our experience is that there is considerable variability in this regard. As Michalos writes, citing Lewis (1946), “because people evaluate things in different ways as they have different experiences, it is impossible for anyone to know exactly what their most appropriate or correct evaluation of anything is, all things considered, until all their experiences are completed.” Our point here is that while subjectivity as to what is ethical enters into all decisions, the creativity inherent in the arts adds an additional dimension of complexity in decision-making regarding ethical research conduct that merits special consideration.

#### Ethics of Authorship and Ownership of Arts-Based Intervention Products

The process of creative collaboration leading to co-creations, such as collaborative devised theatre, visual arts or musical creations, for example, raises questions relating to authorship, ownership, and consent to use the work in multiple venues. Clearly if the research is simply studying an artistic work that is being created by a community-based NGO or participating artist, the group or individual creating the art has full rights. However, when the research grant is actually funding the development of the artistic work, complications arise when either the artist(s) or researcher(s) are based at institutions that may expect to have ownership of the final product. Indeed even if co-ownership is acknowledged by the university and community partner, arguably approval should be sought from both parties involved in the creation before researchers and/or community partners discuss or even cite the work. If for example, the NGO or participating artist wishes to remount the play or exhibit drawings in a public arena, should the NGO or participating artist need approval from the research institute that housed the research grant? Given all the potential areas of conflict and disciplinary differences around the subjects of ownership and authorship (Brydon-Miller 2012), these issues are best addressed prior to launching the research project.

Again, we recognize that there has been considerable scholarship on individual and joint intellectual property rights.(Belderbos et al. 2014; Kanwar 2012; Okamuro and Nishimura 2013), as well as the rights of the individual artist/inventor (Zvulony and Co 2010). However these issues are not always seen as ethical issues. Like Cox and Boydell (2015), we argue that ownership rights need to be part of ethical frameworks for research involving the arts. While we have not yet encountered an ethical *conflict* in this area, it is a consideration that we have come to recognize as a potential concern as the creation of arts-based intervention and/or knowledge mobilization products (including theatrical performances, dances, websites, photos and videos) are a key component of our research project.

#### Right of Acknowledgement Versus Protection of Anonymity

Another issue that elicited heated responses in our questionnaire and that has been a point of conversation was the tension that can emerge between the goals of anonymity and protection of vulnerable participants on the one hand, and the desired goal of promoting the opportunity for individuals and communities to “stand up and speak out.” Further, participants sometimes want their identity to be known, as they are proud of their contribution and want the works they produce and insights they offer to be openly attributed to them, a concern also noted by Cox and Boydell (2015). However, in our experience and survey results, the majority of respondents were in favor of having anonymity as the de facto responses of REBs as a necessary and welcomed safeguard. One questionnaire respondent eloquently discussed the tensions surrounding issues of confidentiality: “…*the consequences of being named in some research projects may have impact that can*’*t be foreseen either by researcher or participant*…*so not naming ensures some protection*…*so there is the tension between honoring and making visible the co*-*researchers that participants are*, *and shielding them from unwanted attention or consequences*....”

Nonetheless, some provision could be made, in situations deemed low risk by ALL parties (including the artist, participants, and community partners), that, with written informed consent, the researchers might identify the artist-participants involved, for example, listing the community members who comprised the cast of a community theatre event or dance group that was the subject of ASC research, or the photographer in a photo-facilitated workshop. We are, for example, currently in the process of negotiating with the senior community artists in our collaboration for permission to include our interviews with them on the web, as a means of honoring their contributions, wisdom and experience, and simultaneously, ensuring broad knowledge dissemination. Despite their initial signing of the consent form, ethically, we felt it incumbent upon us to reconfirm their assent upon viewing their individual interview. Ethical issues of ownership, censorship, acknowledgement of authorship, form of representation, and implications of website publication have all been troubling the process. While there is no one prescription that fits all contexts, we conclude that addressing the issue of acknowledging authorship for creative work and providing protection of anonymity merits consideration as an ethical issue.

#### Ethics of “Dangerous Emotional Terrain” (Gray et al. 2003)

ASC projects often address difficult, emotionally charged, risky and traumatic issues that have the potential to lead to crisis. In our main field projects - with urban youth with marginalized trajectories, individuals with disabilities, those suffering from Parkinson’s disease, and refugee - such situations did arise. While ASC projects often do not set forth a series of specific objectives, by definition they seek to “undo” and encourage participants to see anew the status quo and habits of engagements (Frantzich 2013). As such, like all participatory action research, participation can disrupt what is known, and invite participants and researchers to reconsider their ways of being and engaging in the world (Fels 2011, 2012; Fels et al. 2011). Participation in ASC projects can be unsettling, yet simultaneously liberating. Use of arts-based research methods, or even studying ASC projects using conventional research processes of quantitative or qualitative data collection through surveys, focus groups and interviews, may further provoke this re-thinking. This “undoing” of our habitual engagement speaks to liminal spaces of interrelationships where the “endless dance of co-emergence” (Waldrop 1992) gives rise to new understanding and perspective.

The embodied and deeply emotional nature of the arts and accompanying responsibilities of witnessing makes this issue one that is receiving increasing attention in research involving artists and community partners (Salverson 2008; Boydell et al. 2015). As one questionnaire respondent stated: “*Have clear protocol of work*… *do not work alone*, *do not improvise work with specialist in social field*… *and don*’*t provoke just for the fun of it*, *do it with a plan*…”. Whether a trained specialist (psychologist, social worker, etc.) is actually on site during the art activity, or arrangements are made for contact and quick referral if necessary, our experience suggests that researchers and ethics boards would be remiss to neglect these issues surrounding the well-being and emotional care of participants and in some cases researchers as well. Some guidance in this regard has been offered by Boydell and colleagues (2015), who identify specific strategies to tackle emotional impact in their recent examination of the issue of ‘dangerous emotional terrain’. Significantly, it is important to heed the cautions articulated, for example, in Merli’s (2002) critic of Mataraso’s (1997) opus on evaluating ASC projects – namely the need to guard against preconceived notions of what abstract concepts such as “happiness”, “empowerment” and “confidence” mean to participants; promoting acculturation of participants to one’s own ideas; and judging other people’s quality of life according to the researcher’s own worldview. Clearly in quantitative research, when terms have to be defined for purposes of measuring change, we attempt to use validated scales, for example, for concepts such as “personal growth”(Robitschek 1998) or “social inclusion” (Huxley et al. 2012), an indeed we have used such surveys in several of our ASC! projects (for example, see JB. Spiegel 2014 and JB. Spiegel et al. 2015). Nonetheless, we believe that ensuring that all questions in interviews and survey are posed in a manner that avoids being judgmental is an ethical imperative.

#### Ethics of Representation

There is a long dark history of researchers not only exploiting marginalized populations directly for their own career ambitions, but also representing such communities or individuals in ways that may be disempowering – inadvertently or otherwise – in visual or text descriptions (Flicker et al. 2007) or in role plays (JB. Spiegel and Yassi 2007). This ethical issue of representation, such as poverty porn (Hester 2014), is of particular concern with regards to ASC research, because of the greater use of visual imaging in the arts (in performing arts such as theatrical productions, music, circus, dance, as well as in film, videos, photos, sculptures, drawings, paintings and the like). Eve Tuck in her “damage-centered theories of change” (Tuck 2009), acknowledges that this representation of participant or community as victim, oppressed, unable to take action, is often done with good intentions but is based on what she calls a “flawed theory of change” that suggests that if the extent of damage to a community can be documented, it will be addressed.

Ethical issues can arise when focus is given only to aspects that can be easily dramatized, or if processes do not exist to allow research participants to challenge the interpretation presented. Some argue that leaving the art created in an ASC project open to a greater level of interpretation by the researchers results in a product that may be less ‘true’. Others argue that multiple interpretations enrich the learning that may arise from the data; arts-based research in particular invites multiple perspectives, approaches, and insights to “enlarge the space of possibility” (Sumara and Davis 1997).

Issues of misrepresentation (S. M. Cox and Boydell 2015) and ethical implications of divergent interpretations percolate throughout artistic-academic collaborations. On the issue of the ethics of representation one participant responded, “*Requires that artist*-*researchers provide written discussion of how they are wrestling with this issue and possible strategies. Be sure those in position to create and disseminate the representations are thinking about the risks in a reflective way*.” We echo this advice and, in particular, the need to be reflexive. In addition, our experience suggests that the intent of individual team members or partners in a multi-partnered research project may come into conflict when representation and knowledge dissemination are being considered; we therefore believe that it is useful to consider this issue as an ethical priority.

### Team Issues

#### Ethics of Caring for Team Members, Students and Staff

The main public research granting agencies in Canada do not allow funding of the principal investigator or co-investigators: “Non-Eligible Expenses: Any part of the salary, or consulting fee, to the grantee or to other persons whose status would make them eligible to apply for grants from the Agency.” (Canadian Institutes of Health Research et al. 2014). The assumption is that universities pay adequate salaries to those who lead such projects. Just as the first point we identified under community-university partnership issues above was “inequities in remuneration between university and community research partners”, here we flag the inequities within the university-based team itself. While we recognize that such issues transcend the university’s salary structure, we feel that it is time to recognize that grossly unequal remuneration creates power dynamics (Sultana 2007) that can undermine interdisciplinary university-community research.

With the increasing precariousness of employment in universities, and the increased percentages of short term, part-time and adjunct faculty making up university academic personnel, we urge research teams to be generous as part of an ethical commitment of care for team members, regardless of the fact that existing levels of unemployment means that universities can get away with paying wages to some faculty members that are very low in some categories. Our experience suggests that a research team should assume an ethical responsibility to do as much as possible to take care of the material and emotional needs of all research participants, including poorly-paid project leads or trainees paid on the research grant, as part of the ethical necessity to address power dynamics in the conduct of the research. Just as fulfilling commitments to community partners is an ethical imperative, as discussed above, we consider that fulfilling financial commitments to team members when faced with bureaucratic barriers should be seen in the same light; team managers need to be willing to confront problematic “established institutional protocols” when seeking to address issues of parity or perceived fairness. Administrative protocols can support a team member’s authority/stature within her own institution, and a team can manage the tensions between attending to administrative requirements and devising creative solutions to address the needs of individual researchers and/or the project as a whole. To us, this critical creativity of response and problem-solving on behalf of the research project is part of “walking the talk.” And just as solidarity and social justice are increasingly recognized as part of ethical practice in working with colleagues from low and middle income countries (A. Yassi et al. 2013), as researchers in arts for social change, we consider it crucial that researchers promote solidarity and social justice within a research team itself as a matter of ethics.

#### Ethics of Researcher Engagement and Commitment

We often do not anticipate how deeply invested we are in the research projects in which we are engaged with our community partners. Yet in the words of Cox and colleagues: “[t]his blurring of boundaries creates ethical challenges, such as how to best exit from the project when participants have invested deeply in building relationships and contributing to the research” (S. Cox et al. 2014). Additionally, in collaborative research projects, different researchers shoulder distinct responsibilities, and contribute diverse levels of engagement, commitment, and time. Sometimes these varying levels of responsibilities are known from the start; sometimes work or family commitments outside the project alter originally envisioned commitments; and sometimes researchers become disenchanted with the research and “vote with their feet” devoting less time to a project. How a collaborative research group negotiates, navigates, and resolves issues arising from perceived or actual differences in individual engagement and commitment to the project has ethical dimensions. Here we are governed by compassion, and invitation to individual researchers to engage at levels that are most appropriate given their current situations and responsibilities, but our experience underlines the fact that if one researcher does not fulfil the responsibilities taken on, this impacts others across the team – which itself has ethical dimensions.

Another set of issues identified in the survey and which we have experienced were the emotional, logistical and ethical issues that arise when a specific individual with whom the researchers built a relationship leaves the community partner organization. Many university-community partnerships are forged on relationships, and when someone leaves an institution it may be difficult to salvage the research partnership. Moreover, in acrimonious departures, the dilemma often arises as to whether the researchers should remain loyal to the organization and try to continue the work with the successor to the original collaborator, or rather maintain the research relationship with the individual with whom the relationship was established if the individual’s new role permits this. Over a short two and a half year period, we have seen major players exit, change institutions, assistant researchers graduate, and/or refocus on their own studies, all at a perceived or possible cost to the research project.

Thus our reflections indicate that in order to sustain multi-year research projects, firm, clear, written protocols concerning roles and responsibilities are desirable, including not only budget creation and monitoring, but also protocols when personnel changes occur. These could include communication strategies, both internally and externally. While we cannot guarantee that identifying and confirming process protocols and responsibilities at the outset of the project will prevent confusion about expectations, our group has identified this type of activity as a necessary ethical priority because of the potentially negative consequences of failing to pro-actively address such issues.

#### Ethics of Expanding the Team after the Project is in Process

Research is increasingly becoming a collective endeavour, and, as encouragement for larger and more interdisciplinarity on teams increases (Novak et al. 2014), so too does the need, and indeed desire of researchers and artists to seek to co-research and co-create with colleagues, friends, and those with whom there is a shared recognition of theoretical resonance, expertise, perspective and previous lived experience. Concerns may arise when, in the course of discussing the research with friends, colleagues and family members, an idea is generated to expand the team to include the expertise of such individuals. This preference is, of course, not unique to research projects – we all prefer to work with people with whom we enjoy working, and it would be foolish to want to do otherwise. As our friends, family or colleagues learn about our endeavours, they may want to join in, or we may want to include them when we realize the added insight such people could contribute. Inviting friends, family and colleagues to join an existing collaboration can be tricky. Our team has several kinship and longstanding friendships amongst the original group of team members, yet we have to date avoided bringing in *additional* colleagues, family or friends as co-investigators in order to avoid interfering with the already established bonds of trust amongst team members whose roles within the team could be inadvertently destabilized by introducing new researchers.

However, there are occasions when new researchers are required to join to team (when others depart, research assistants graduate, or new requirements are identified). Before contemplating such steps, our work to date indicates that it is critical to ensure that doing so does not undermine potential contributions of the original research team members or skew the original intent of the research. Engaging new members into an existing research team requires care, oversight, and responsibility. As team relations are so important for successful collaborative research, how to add members to an established team is an ethical issue worth including while addressing the previously described issue of protocols for commitment and engagement.

#### Ethics of Respect for Interdisciplinarity – Different Cultures of Publication, Collaboration, and Notions of Ethical Practice

Compounding the ethical challenges identified above is the issue of interdisciplinarity - an essential characteristic of ASC research. Ethical dilemmas may arise as different disciplines draw on diverse languages, perspectives, processes, and values in terms of what matters, whose authority is to be respected, ways of engagement, and how to negotiate through difficult tensions such as team decision-making, reporting, procedural processes and especially authorship. Our experience revealed that invitation to co-authorship is a particularly complex issue in large multi-partner research teams. In the humanities and education, greater value is granted in academia to single author papers, while in the health sciences, multiple authors is a standard. Indeed the percent of single-authored, or even dual-authored, publications in some science fields is becoming rare. Lewis et al. (2012), in reviewing publishing patterns, reported that almost no scientist in their study published alone, while this was not the case in the arts and humanities (Lewis et al. 2012). The circumstances meriting offer to co-author has been a source of tension within our team: Should those of us who are leading the writing of an article about this team project invite all other team members to contribute as co-authors? Such a generous gesture would result in a long listing of author names, and extensive time-consuming consultation on issues on which some team members may have only limited knowledge or interest. Moreover, there is a bigger issue here. Multi-authored papers would also compromise the humanities authors’ capacity to develop a nuanced analysis drawing on the specialized theoretical literature of their field; others will be informed by different theoretical literatures and debates thus potentially watering down the argument or pulling it in radically different directions such that it would no longer be pertinent to the initial conversation to which the lead author wished to contribute.

This concern of competing interests and modes of authorship extends to community partners; there are multiple goals of research outputs, reflecting different values and interests. Generosity in extending invitation to co-author requires different answers in different disciplines, making interdisciplinary decisions complex. Our experience underlines the importance for teams of artists, community activists and scholars from different disciplines to openly discuss the varied needs with respect to authorship as part of considering ethical practice in the research endeavor.

Even more important, perhaps, are the disciplinary differences in determining what is ethical research practice itself. Many qualitative researchers are interested in studying the lived experiences in a given setting, taking great care not to intervene; some critical scholars have even become reluctant to engage in fieldwork at all for fear that this type of research could be exploitative research perpetuating relations of domination and control. In contrast, health researchers interested in evaluating the effectiveness of ASC interventions often believe that intervention studies—preferably cluster randomized controlled trials (RCTs)—are the most ethical way to proceed, if feasible to do so, as these would produce the most robust results accordingly to this framework of the hierarchy of evidence. Indeed cognizant of the ethical violation of “drive by data collecting” in which problems are identified but not addressed, some researchers on our team felt that it is unethical not to try to design, implement and evaluate ASC interventions using the most rigorous protocols available, including RCTs if budgets allow and the timeframe is such that it is feasible to do so (A. Yassi et al. 2014). Unfortunately, constraints on the part of the community partners precluded the implementation of an RCT within the ASC! partnership, and we have had to be content with observations and other forms of research, rendering this debate moot in our case.

Interdisciplinary differences regarding what is ethical research are particularly poignant when it comes to evaluating ASC projects. Stein and Faigin (2015) noted that “when art activities are framed in terms of their capacity to…‘fix the problems’ of people identified by the dominant culture as ‘deficient’ or ‘at risk’, there is the danger that the arts simply become an instrument for perpetuating oppression and the status quo” (Stein and Faigin 2015). Yet, as we mentioned above, quantitative researchers tend to be drawn to measurable indicators that do just that. Merli (Merli 2002) argued in her critique of Matarasso (Matarasso 1997) that the empirical research he seems to have done offers nothing to counter the imposition of the researcher’s values and ideas on participants. The concern here is that administering questionnaires with pre-determined indicators, such a personal growth and social inclusion, as we noted above, in order to evaluate community-based arts initiatives can merely reinforce dominate cultural stereotypes and perpetuate existing definitions of social problems. And, qualitative researchers on our team have often noted, quantitative research is generally predicated on the use of exactly such indicators with their embedded socially desirable assumptions. Meanwhile, standing eager on the sidelines are arts-based researchers with their own understanding, theories, and practices of inquiry. Acknowledging such interesting differences is essential to ethical practice. How does a multi-partnered multi-disciplined research partnership respectively navigate the various and potentially contentious ethical contradictions inherent within the work? In our case, we did indeed combine quantitative and qualitative methods in at least three of our field studies, with the team agreeing that the combination strengthened the research, the epistemological challenges notwithstanding. We found overall that critical reflection within the team on the different normative assumptions inherent in research designs - including but not limited to the quantitative scales employed, the respondents’ understanding of certain terms (e.g., sense of accomplishment, sense of community, feeling accepted), as well as the goals of the community art interventions themselves - is itself an ethical priority.

## Discussion and Conclusion

While existing literature outlines ethical issues of university-community partnership (Gelmon et al. 1998; Seifer and Carriere 2003; Baum 2000; Andrews et al. 2012; Northmore and Hart 2011), no study to our knowledge unpacks the ethical issues that arise when university-community partnerships involve not only health, education, and social sciences, but researchers from the arts and humanities as well. Moreover, there is a lack of discussion of the ethical issues pertaining not only to the “treatment of research subject” but rather to the larger and often highly contentious concerns of “ethical practice in a research team’s functioning”. These issues are inextricably linked. If the community partners’ concerns are not respected, it will undermine the research and ultimately the wellbeing of research team members as well as contribution to world knowledge. The converse is also true; tensions within the research team will impact the quality of the research (Curry et al. 2012). As such, we argue that it is ethically imperative to develop a plan sensitive to the power dynamics within the community-artist-researcher team. To do so requires that researchers be reflexive in relation to interpersonal aspects of the research process, not just the epistemological aspects (Guillemin and Gillam 2004). Once again, drawing on Barry et al.’s (1999) framework for optimizing team functioning through reflexivity had proven a valuable resource for our team.

Hopefully the issues raised in this paper will be useful for teams engaged in these types of partnership, encouraging discussion in the early stages and using reflexive dialogue to arrive at a context-specific collaborative approach for each issue. However, we also realize that we need to remain willing to be surprised, to rally together to renegotiate, to fail so as to learn, to navigate, to undo what is known, and to engage in what we have not yet imagined. This reflexive dialogue needs to be woven into the tapestry of our ethical responsibilities to each other, our partners, and our participants. We have an ethical responsibility to be wide-awake (Greene 1978), and mindfully aware, attentive to the tugs on the sleeve (Fels 2012), the stops (Appelbaum 1995), those moments that unsettle, that call us to attention.

Arts–for-social-change research is, by definition, intended to generate knowledge, attitudes and skills that promote social change. In our questionnaire we asked: Are researchers adequately considering their commitment to building capacity for social change in the communities in which they are working? We contend by closely examining our own practices, relationships, and willingness to identify areas of ethical conflict, tension and gaps, that we are learning how to identify areas required for social change within our research partnerships, our institutions, our practices, and our communities. Michalos observed that discussion of most ethical appraisals, especially in committees, is generally very shallow and that “deep disagreements about moral appraisals make people uncomfortable, require more time, attention and work, threaten individual belief systems and self-esteem, and put group solidarity and sustainability at risk.” (Michalos 2001). Nonetheless, in terms of producing research able to fully serve the needs and respect the contributions of all involved in our project, engagement in the ethical concerns and issues of a proposed research initiative, particularly one that is multi-partnered and interdisciplinary, indeed requires thoughtful attention and consultation among all parties involved.

Sultana, analyzing her own work with communities in Bangladesh related to water access, calls on researchers to be aware of “issues of reflexivity, positionality and power relations in the field in order to undertake ethical and participatory research.” (Sultana 2007). We feel this is key for all research teams, but especially those working in interdisciplinary teams involving artists and communities, in order to achieve more ethical research practices. However, we would like to go farther; notwithstanding serious challenges imposed by the diversity of disciplinary traditions, as discussed in “Discussion and conclusion” section, we argue that it is not enough to reflect on power relations – it is an ethical necessity that we seek to address these in how we actually conduct research in the field of arts-for-social-change.

An astute reader may wonder about the gaps and tensions, hinted at, as yet unspoken, in our own evolving relationships of engagement and inquiry. Somehow it is not surprising that we find ourselves, together, seeking illumination and resolution in the ethics of engagement as we embark upon our multi-disciplinary multi-partnership adventure. As educator Madeline Grumet advised, *we must tread lightly*, *oh so lightly*,^1^ as we lay down our path in walking (Varela 1987) in a landscape unfamiliar, unsettling, challenging. As educators and researchers, artists, participants and community workers, as learners, we are invited to listen, to be compassionate and ethically attend to all of the possibilities that await us (Fels 2012). We thought we had already identified ethical challenges before our project started; what we learned is that given the complexity and diversity of team membership and projects, ethical issues are emergent and require attention on an ongoing basis. Sultana (2007) argued that: “ethical research is produced through negotiated spaces and practices of reflexivity that is critical about issues of positionality and power relations at multiple scales.” She noted that “[a]ttempts to institutionalize ethical frameworks are not sufficient to address or ensure good practice in the field. There are critical disjunctures between aspects of everyday behaviour in the field and the University’s institutional frameworks that aim to guide/enforce good ethical practice, as the very conduct of fieldwork is always contextual, relational, embodied, and politicalized” (Sultana 2007). Guillemin and Gillam (2004) agree, noting: “Research ethics committees cannot help when you are in the field and difficult, unexpected situations arise, when you are forced to make immediate decisions about ethical concerns, or when information is revealed that suggests you or your participants are at risk” (Guillemin and Gillam 2004). What we offer, stopping at the mid-way station in our research, is a map of lessons learned so far from such critical conjunctures in our large interdisciplinary community-university- artists research program, so that others, researchers, post-secondary graduates, and educators, need not stumble in our footsteps, as they pursue their own path of creating and navigating ethical, responsible, caring research.

## Footnotes

1 Borrowed from the title of a presentation by Madeleine Grumet, University of British Columbia, Vancouver, Canada, n.d.
